# Influence of Coding Variability in APP-Aβ Metabolism Genes in Sporadic Alzheimer’s Disease

**DOI:** 10.1371/journal.pone.0150079

**Published:** 2016-06-01

**Authors:** Celeste Sassi, Perry G. Ridge, Michael A. Nalls, Raphael Gibbs, Jinhui Ding, Michelle K. Lupton, Claire Troakes, Katie Lunnon, Safa Al-Sarraj, Kristelle S. Brown, Christopher Medway, Jenny Lord, James Turton, ARUK Consortium, Kevin Morgan, John F. Powell, John S. Kauwe, Carlos Cruchaga, Jose Bras, Alison M. Goate, Andrew B. Singleton, Rita Guerreiro, John Hardy

**Affiliations:** 1 Reta Lila, Weston Research Laboratories, Department of Molecular Neuroscience, UCL Institute of Neurology, London, United Kingdom; 2 Laboratory of Neurogenetics, National Institute on Aging, National Institutes of Health, Bethesda, MD, United States of America; 3 Department of Experimental Neurology, Center for Stroke Research Berlin (CSB), Charite’ Universitätmedizin, Berlin, Germany; 4 German Center for Neurodegenerative Diseases (DZNE), Berlin site, Germany; 5 Departments of Biology, Neuroscience, Brigham Young University, Provo, UT, United States of America; 6 King's College London Institute of Psychiatry, London, United Kingdom; 7 QIMR Berghofer Medical Research Institute, Brisbane, Queensland, Australia; 8 Translation Cell Sciences-Human Genetics, School of Life Sciences, Queens Medical Centre, University of Nottingham, Nottingham, United Kingdom; 9 Washington University, Division of Biology and Biomedical Sciences St. Louis, MO, United States of America; 10 Icahn School of Medicine at Mount Sinai, Icahn Medical Institute, New York, NY, United States of America; National Center for Geriatrics and Gerontology, JAPAN

## Abstract

The cerebral deposition of Aβ_42_, a neurotoxic proteolytic derivate of amyloid precursor protein (APP), is a central event in Alzheimer’s disease (AD)(Amyloid hypothesis). Given the key role of APP-Aβ metabolism in AD pathogenesis, we selected 29 genes involved in APP processing, Aβ degradation and clearance. We then used exome and genome sequencing to investigate the single independent (single-variant association test) and cumulative (gene-based association test) effect of coding variants in these genes as potential susceptibility factors for AD, in a cohort composed of 332 sporadic and mainly late-onset AD cases and 676 elderly controls from North America and the UK. Our study shows that common coding variability in these genes does not play a major role for the disease development. In the single-variant association analysis, the main hits, none of which statistically significant after multiple testing correction (1.9e^-4^<p-value<0.05), were found to be rare coding variants (0.009%<MAF<1.4%) with moderate to strong effect size (1.84<OR<Inf) that map to genes mainly involved in Aβ extracellular degradation (*TTR*, *ACE*), clearance (*LRP1*) and APP trafficking and recycling (*SORL1*). These results were partially replicated in the gene-based analysis (c-alpha and SKAT tests), that reports *ECE1*, *LYZ* and *TTR* as nominally associated to AD (1.7e^-3^ <p-value <0.05). In concert with previous studies, we suggest that 1) common coding variability in APP-Aβ genes is not a critical factor for AD development and 2) Aβ degradation and clearance, rather than Aβ production, may play a key role in the etiology of sporadic AD.

## Introduction

The cerebral deposition of Aβ_42_ aggregates, insoluble neurotoxic derived of amyloid precursor protein (APP), is likely caused by an imbalance between Aβ production and clearance and represents a key event in Alzheimer’s disease (Amyloid hypothesis) [1].

A growing body of evidence has pointed to the critical role of APP-Aβ metabolism in AD pathogenesis. First, the discovery of *APP*, *PSEN1* and *PSEN2* mutations showed that familial Alzheimer’s disease is linked to Aβ_42_ overproduction [2–4]. Second, genome-wide association studies (GWASs) identified several susceptibility loci associated with AD (*APOE*, *BIN1*, *PICALM*, *CD33*, *ABCA7*, *CLU*, *MS4A6A*, *EPHA1*, *CR1*, *CD2AP*, *SORL1*, *CASS4*) and regulating APP-Aβ levels [5–12]. Third, next generation sequencing laid the ground for the discovery of *TREM2* risk variants, highlighting the possible role of microglia in Aβ clearance [13–14]. Finally, although recent studies have shown that rare coding variability in *PSEN1* may influence the susceptibility for apparently sporadic late-onset AD (LOAD) [15–16], increases in Aβ production currently explain a minority of AD cases. By contrast, it is very likely that the majority of AD cases are caused by impaired degradation and clearance of Aβ, which is produced at normal levels throughout life [17–18]. Despite the importance of APP-Aβ metabolism in AD, the role of genes taking part in Aβ production and catabolism as susceptibility factors for AD is still elusive and has not been extensively investigated. Therefore, in this study, we selected 29 genes known to be involved in APP and Aβ processing: *ADAM9*, *ADAM10*, *ADAM17*, *MEP1B*, *BACE1*, *BACE2*, *NCSTN*, *PSENEN*, *APH1B*, *LRRTM3*, *APLP1*, *APBA1*, *SORL1*, *TTR*, *GPR3*, *ECE1*, *ECE2*, *IDE*, *CST3*, *CTSB*, *CTSD*, *LYZ*, *MME*, *ACE*, *MMP3*, *A2M*, *PLAT*, *KLK6* and *LRP1*. We then analyzed the single, independent and the cumulative effect of protein coding variants in these genes from exome and genome sequencing data, in a cohort composed of 332 sporadic and mainly late-onset AD cases and 676 elderly controls from North America and UK.

## Materials and Methods

We used exome and genome sequencing data to identify common, low frequency, and rare coding variants in 29 genes involved in: Aβ production (*ADAM9*, *ADAM10*, *ADAM17*, *BACE1*, *BACE2*, *NCSTN*, *PSENEN*, *APH1B*, *MEP1B*, *LRRTM3*, *GPR3*), APP stabilization (*APLP1*, *APBA1)*, APP recycling (*SORL1*), Aβ deposition (*TTR*), intracellular degradation (*ECE1*, *ECE2*, *IDE*, *CST3*, *CTSB*, *CTSD*, *LYZ*, *MME)*, extracellular degradation and clearance (*ACE*, *MMP3*, *A2M*, *PLAT*, *KLK6*, *LRP1)*.

These genes were chosen on the basis of PubMed based literature search and/or based on predicted protein interactions using STRING (http://string.embl.de/).

The discovery cohort was composed of 332 apparently sporadic AD cases and 676 elderly controls, neuropathologically and clinically confirmed, originating from the UK and North America. The mean age at disease onset was 71.66 years (range 41–94 years) for cases and the mean age of ascertainment was 78.15 years (range 60–102 years) for controls (**Table 1**). The majority of the cases (77%) were late-onset (> 65 years at onset).

**Table 1:** Cohort description.

COHORTS	N	TYPE	SEQUENCING STRATEGY	ORIGIN		AGE (YRS)	MALE	*APOE*
						MEAN ±SD(RANGE)	(%)	E4+ (%)
**NIH-UCL**								
cases	127	neuropath	Exome sequencing		Caucasian (British)	65.5(41–94)	46.4	58
controls*	204	neuropath	Exome sequencing		Caucasian (British, North American)	79.8 (61–102)	58.3	45
**WashU**								
cases	23	clinical	Exome sequencing		Caucasian(North American)	57 (46–75)	52.17	NA
controls	16	clinical	Exome sequencing		Caucasian(North American)	79.5 (75–92)	43.7	NA
**ADNI**								
cases	182	clinical	Exome sequencing		Caucasian(North American)	74.65 (55–90)	67	56.6
controls	257	clinical	Exome sequencing		Caucasian(North American)	74.68 (60–90)	50.1	27.6
**BYU**								
controls	199	clinical	Genome sequencing		Caucasian(North American)	80.8 (75–94.5)	37.7	100

NA, not available; YRS, years.

*These controls have been largely used in 2 previous studies [19–20]

Among the cases and controls, 42% and 51% were female, respectively. 58% and 47% of the cases and controls carried the *APOE* ε4 allele, respectively. The *APOE* ε4 allele was significantly associated to the disease status in the NIH and ADNI series (p-value = 0.02 and 1.19x10^-9^, respectively). Importantly, all the BYU controls from the Cache County Study on Memory in Aging were heterozygous for *APOE* ɛ4 allele. However, given the fact that 1) they were elderly (mean age 80.8 years old [range: 75–94.59]) and without any clinical sign of dementia and 2) *APOE* ɛ4 allele is a risk but not a causative factor, we still considered them as controls and included in the study.

Written consent for participation was obtained in accordance with institutional review board standards. All samples had fully informed consent for retrieval and were authorized for ethically approved scientific investigation. The UCLH Research Ethics Committee number 10/H0716/3, BYU IRB, Cardiff REC for Wales 08/MRE09/38+5, REC Reference 04/Q2404/130, National Research Ethics Service (NRES) specifically approved this study.

### Exome sequencing

We performed whole exome sequencing on a cohort of 332 sporadic and mainly late-onset AD cases and 477 elderly controls. DNA was extracted from blood or brain both for cases and controls using standard protocols. Library preparation for next generation sequencing used DNA (between 1 μg and 3 μg) fragmented in a Covaris E210 (Covaris Inc.). Following fragmentation, DNA was end-repaired by 5’phosphorylation, using the Klenow polymerase. A poly-adenine tail was added to the 3’end of the phosphorylated fragment and ligated to Illumina adapters. After purification using an AMPure DNA Purification kit (Beckman Coulter, Inc), adapter-ligated products were amplified. The DNA library was then hybridized to an exome capture library (NimbleGen SeqCap EZ Exome v2.0, Roche Nimblegen Inc. or TruSeq, Illumina Inc.) and precipitated using streptavidin-coated magnetic beads (Dynal Magnetic Beads, Invitrogen). Exome-enriched libraries were PCR-amplified, and then DNA hybridized to paired-end flow cells using a cBot (Illumina, Inc.) cluster generation system. Samples were sequenced on the Illumina HiSeq™ 2000 using 2x100 paired end reads cycles.

### Whole Genome sequencing

Genome sequencing was performed in 199 elderly, clinically healthy controls, from the Cache County Study on Memory in Aging. DNA was extracted from blood using standard protocols. All samples were sequenced with the use of Illumina HiSeq technology. Alignment was performed with the use of CASAVA software and variant calling was performed with the use of SAMtools [21] and the Genome Analysis Toolkit GATK [22]. This sequencing and variant calling were performed by our collaborators at Brigham Young University.

### Bioinformatic

Sequence alignment and variant calling were performed against the reference human genome (UCSC hg19). Paired end sequence reads (2x100bp paired end read cycles) were aligned using the Burrows-Wheeler aligner [23]. Format conversion and indexing were performed with Picard (www.picard.sourceforge.net/index.shtml). GATK was used to recalibrate base quality scores, perform local re-alignments around indels and to call and filter the variants [22]. VCFtools was used to annotate gene information for the remaining novel variants. We used ANNOVAR software to annotate the variants [24]. Variants were checked against established databases (1000 Genomes Project and dbSNP v.134). The protein coding effects of variants was predicted using SIFT, Polyphen2 and SeattleSeq Annotation (gvs.gs.washington.edu/SeattleSeqAnnotation). All variants within the coding regions of 29 candidate genes (*A2M* [NM_000014], *ACE* [NM_000789], *ADAM9* [NM_003816], *ADAM10* [NM_001110], *ADAM17* [NM_003183], *APBA1* [NM_001163], *APH1B* [NM_031301], *APLP1* [NM_001024807], *BACE1* [NM_012104], *BACE2* [NM_012105], *CST3* [NM_000099], *CTSB* [NM_001908], *CTSD* [NM_001909], *ECE1* [NM_001397], *ECE2* [NM_014693], *GPR3* [NM_005281], *IDE* [NM_004969], *LRP1* [NM_002332], *KLK6* [NM_001012964], *LRRTM3* [NM_178011], *LYZ* [NM_000239], *MEP1B* [NM_005925], *MME* [NM_000902], *MMP3* [NM_002422], *NCSTN* [NM_015331], *PLAT* [NM_000930], *PSENEN* [NM_172341], *SORL1* [NM_003105], *TTR* [NM_000371]) have been collected and analyzed. (**S1 Table**) (Further details are provided in the supplementary materials)

### Statistical Analysis

For each variant, allele frequencies were calculated in cases and controls and Fisher’s exact test on allelic association was performed. All computations were performed in R (version x64 3.0.2, http://www.r-project.org/). The threshold call rate for inclusion of both subjects and variants in the analysis was 95%.

In this study, we have sufficient power (≥80%) to detect common SNVs (MAF > 5%) with modest effect (OR = 2) through single-variant association analysis; however, we had limited power (<80%) to detect very rare SNVs (MAF < 0.1%), even those with strong effect (OR > 4).

A p-value of 0.05 was set as a nominal significance threshold. Based on simple Bonferroni correction for multiple testing, the thresholds for single variant and gene-based association are defined by p-value = 1.9e^-4^ (0.05/256 coding variants) and 1.7e^-3^ (0.05/29 genes), respectively.

For the gene-based analysis we have pooled together coding and non-coding variants with a MAF ≤0.05 and studied their cumulative effect on the AD trait.

C-alpha test and SKAT are closely related, being both non-burden tests, analyzing and collapsing the effect of genetic variants of different frequency (common and rare), effect (protective, damaging and neutral) and effect size (modest, moderate, strong). SKAT can be considered an expansion of the c-alpha test because overcomes some of its limits. Indeed, SKAT 1) can be applied also to the study of continuous traits; 2) does not need any permutation; 3) applies covariates to the study. In addition, we have used a set of 5–10 size matched genes not linked to APP-Aβ metabolism (based on a Pubmed and STRING search) as negative controls for the gene-based association analysis. Moreover, we have assessed the reliability of our results, comparing the total variant frequency of the selected genes in our study with the one reported for the European-American cohort in the Exome Variant Server (EVS)(http://evs.gs.washington.edu/EVS/).

## Results

The study population consisted of a total of 332 sporadic and mainly late-onset AD cases and 676 elderly controls of British and North American ancestry (**Table 1**).

We do not report any pathogenic mutation in *APP*, *PSEN1* and *PSEN2* in our cohort. However, one of the controls was an heterozygous carrier of the protective variant *APP* p.A673T (MAF 7x10e^-4^ in our cohort and MAF 5x10e^-4^ among the European non-finnish, ExAC database, released 13 January 2015).

We performed a single-variant and a single-gene association analysis in a pre-defined set of genes involved in APP processing (*ADAM9*, *ADAM10*, *ADAM17*, *MEP1B*, *BACE1*, *BACE2*, *NCSTN*, *PSENEN*, *APH1B*, *APLP1*, *APBA1)*, Aβ metabolism and catabolism (*LRRTM3*, *LRP1*, *TTR*, *GPR3*, *SORL1*, *ECE1*, *ECE2*, *IDE*, *CST3*, *CTSB*, *CTSD*, *LYZ*, *MME*, *ACE*, *MMP3*, *A2M*, *PLAT*, KLK6), including 68 Megabase pairs (Mbs) of coding sequence.

A total of 960 single nucleotide variants (SNVs) has been identified. Among these, 256 (26.6%) were nonsynonymous, 194 (20.20%) were synonymous, 97 (10.1%) were intronic and 413 (43%) UTR variants. Among the missense variants, 192 (75%) were very rare (MAF<1%), 16 (6.25%) were low frequency (1%<MAF<5%) and 12(4.68%) were common (MAF>5%). In addition, we report 36 novel coding variants. Variant minor allele frequency and novel variants were based on ExAC database, released 13 January 2015, or dbSNP 137(**S2 Table**). The overall variant frequency in our cohort was in line with the variant frequency reported in the American-European cohort in the Exome Variant server database (**S3 Table**).

Moreover, 120 missense variants (46.8%) were described as damaging variants by at least 2 out of 3 *in silico* prediction softwares (SIFT, Polyphen and Mutation Taster). Importantly, genes involved in Aβ degradation and clearance harbor the highest relative frequency of rare coding and damaging variants (mean = 4.73 rare coding variants/Kbp of coding sequence and 3.16 damaging coding variants/Kbp of coding sequence, respectively). By contrast, genes taking part in APP processing and Aβ production, present the lowest relative frequency of rare coding and damaging variants (mean = 3.59 rare coding variants/Kbp of coding sequence and 1.5 damaging coding variants/Kbp of coding sequence, respectively), suggesting a higher degree of conservation of this last cluster of genes (**S4 and S5 Tables**).

### Single coding variant association test

We identified 3 nominally significant variants, clustering in genes involved in Aβ catabolism (*TTR* [p.T139M], *ACE* [p.T916M]) and APP cleavage (*APH1B* [p.T27I]). Overall, the main hits (variants with the lowest p-values) mainly cluster to genes predominantly involved in Aβ degradation and clearance (6 out of 8 genes [75%]), with *ACE*, *SORL1* and *LRP1* harboring multiple variants, compared to APP processing (2 out of 8 genes [25%]). Moreover, most of these top genes are highly expressed in the brain (http://biogps.org/) and harbor very rare coding variants (0.009%<MAF<1.4%) with moderate to strong effect size (1.84<OR<Inf). (**Table 2**).

**Table 2:** Most significant variants.

GENE	POSITION	RS ID	nucleotide change	Aa change	CARRIER AD (%)	CARRIER. CTRLS (%)	SIFT	POLYPHEN	MAF cases-ctrls	ExAC	PVAL	Corrected p-value	OR	OR 95% CI
*TTR*	chr18:29178610	rs28933981	c.C416T	p.T139M	6/332 (1.8)	2/676 (0.29)	deleterious	probably damaging	0.009–0.0014	0.001475	0.018	1	6.19	1.099–63.091
*ACE*	chr17:61568577	rs3730043	c.C2747T	p.T916M	10/332 (3)	8/676 (1.18)	deleterious	probably damaging	0.015–0.006	0.004348	0.0456	1	2.59	0.910–7.631
*APH1B*	chr15:63569902	rs117618017	c.C80T	p.T27I	53/332 (15.9)	144/676 (21.3)	tolerated	benign	0.08–0.106	0.09078	0.051	1	0.702	0.486–1.003
*SORL1*	chr11:121367627	rs117260922	c.G808A	p.E270K	16/332 (4.8)	18/676 (2.6)	deleterious	probably damaging	0.024–0.013	0.01468	0.0936	1	1.849	0.869–3.90
*SORL1*	chr11:121495816	rs140327834	c.A6194T	p.D2065V	0/332 (0)	7/676 (1.03)	tolerated	probably damaging	0–0.005	0.002823	0.102	1	0	0–1.407
*CTSB*	chr8:11704617	rs114308907	c.A737C	p.N246T	3/332 (0.9)	1/676 (0.14)	deleterious	probably damaging	0.004–0.0007	0.001936	0.107	1	6.143	0.491–322.997
*LRP1*	chr12:57606021	rs142605462	c.G13471C	p.D4491H	3/332 (0.9)	1/676 (0.14)	tolerated	probably damaging	0.004–0.0007	0.0005242	0.107	1	6.143	0.491–322.997
*MMP3*	chr11:102711201	rs148047905	c.G749A	p.R250H	2/332 (0.6)	0/676 (0)	tolerated	benign	0.003–0	0.0004121	0.1083	1	Inf	0.382- Inf
*NCSTN*	chr1:160321594	NA	c.G842A	p.R281Q	2/332 (0.6)	0/676 (0)	tolerated	benign	0.003–0	0.00009889	0.108	1	Inf	0.382- Inf
*SORL1*	chr11:121323072	rs147575757	c.G32C	p.R11P	2/332 (0.6)	0/676 (0)	deleterious	benign	0.003–0	0.0002606	0.108	1	Inf	0.382- Inf
*ACE*	chr17:61564428	rs148995315	c.G2299A	p.E767K	2/332 (0.6)	0/676 (0)	deleterious	possibly damaging	0.003–0	0.0003083	0.108	1	Inf	0.382- Inf
*LRP1*	chr12:57522754	NA	c.A7C	p.T3P	2/332 (0.6)	0/676 (0)	tolerated	benign	0.003–0	NA	0.108	1	Inf	0.382- Inf
*LRP1*	chr12:57589676	rs143285614	c.G8591A	p.R2864H	2/332 (0.6)	0/676 (0)	tolerated	benign	0.003–0	0.0001904	0.108	1	Inf	0.382- Inf
*LRP1*	chr12:57587773	rs149099223	c.C7896A	p.D2632E	2/332 (0.6)	0/676 (0)	tolerated	probably damaging	0.003–0	0.0005081	0.1083	1	Inf	0.382- Inf

Position is in hg19/GRCh37. MA, minor allele; CTRLS, controls; MAF, minor allele frequency; OR, odds ratio; inf, infinity; CI, confidence interval. Corrected p-value, p-value after Bonferroni correction (p-value*256 [number of variants considered in the single-variant association test])

However, none of the coding variants detected in the studied genes reached the statistical significance, based on a corrected p-value (p-value <1.9e^-4^), in the single-variant association test.

For all these main variants, with the *APH1B* (p.T27I) and *SORL*1 (p.D2065V) exceptions, the minor allele was substantially more frequent in cases compared to controls, suggesting a possible role as a risk factor for AD. The study possessed relatively low power to detect any significant association between cases and controls for low frequency and rare variants. Nevertheless, we analyzed these variants because we could not preclude the possibility that high effect risk alleles were present.

### Gene-based association test

In addition to single-marker analysis, we performed gene-wide analysis to combine the joint signal from multiple coding and non-coding variants with a MAF≤ 0.05 within a gene and to provide greater statistical power than that for single-marker tests. All the variants (nonsynonymous, synonymous, UTRs) located within the studied genes and their exon-intron flanking regions were collapsed together and their joint effect has been studied and compared with 5 to 10 size and variant matched gene controls (**S6A and S6B Table**). Genes involved in Aβ degradation and clearance were enriched for the lowest p-values. The combined effect of variants in *ECE1*, *LYZ* and in *TTR* reached the nominal significance in the c-alpha and SKAT tests, respectively (1.7e^-3^ <p-value <0.05) (**Tables 3 and 4**). There was a partial overlap between genes identified in the single-marker analysis and those with SKAT and c-alpha tests. Importantly, *TTR* was the main finding both in the SKAT and single-marker association analysis. Therefore, suggesting *TTR* as a promising potential candidate risk gene for AD.

**Table 3:** C-ALPHA TEST.

TRANSCRIPT ID	POSITION	GENE	N.VARIANTS	TEST	P-VALUE	CORRECTED P-VALUE
NM_001397	chr1:21543823..21671981	*ECE1*	51	CALPHA	0.00576606	0.1653
NM_000239	chr12:69742188..69747889	*LYZ*	5	CALPHA	0.0322581	0.928
NM_001908	chr8:11700101..11725587	*CTSB*	75	CALPHA	0.0706522	1
NM_000371	chr18:29171879..29178899	*TTR*	6	CALPHA	0.142857	1
NM_003183	chr2:9629731..9695906	*ADAM17*	18	CALPHA	0.16	1
NM_014693	chr3:183967457..184010734	*ECE2*	32	CALPHA	0.22	1
NM_000789	chr17:61554500..61574779	*ACE*	45	CALPHA	0.193548	1
NM_003816	chr8:38854521..38962660	*ADAM9*	23	CALPHA	0.196429	1
NM_001110	chr15:58888656..59042155	*ADAM10*	26	CALPHA	0.275	1
NM_031301	chr15:63569800..63601264	*APH1B*	39	CALPHA	0.277778	1
NM_000014	chr12:9220607..9268549	*A2M*	26	CALPHA	0.277778	1
NM_012105	chr21:42539814..42648229	*BACE2*	88	CALPHA	0.285714	1
NM_002332	chr12:57522754..57607023	*LRP1*	111	CALPHA	0.322581	1
NM_015331	chr1:160313330..160328428	*NCSTN*	21	CALPHA	0.36	1
NM_001012964	chr19:51462012..51471329	*KLK6*	11	CALPHA	0.444444	1
NM_000902	chr3:154797478..154901245	*MME*	40	CALPHA	0.6	1
NM_012104	chr11:117156543..117186818	*BACE1*	39	CALPHA	0.714286	1
NM_000099	chr20:23614297..23618571	*CST3*	12	CALPHA	0.714286	1
NM_002422	chr11:102706685..102714317	*MMP3*	16	CALPHA	0.714286	1
NM_001163	chr9:72042470..72287163	*APBA1*	41	CALPHA	0.833333	1
NM_178011	chr10:68685929..68860827	*LRRTM3*	29	CALPHA	0.833333	1
NM_172341	chr19:36237681..36237803	*PSENEN*	2	CALPHA	1	1
NM_005925	chr18:29772759..29800324	*MEP1B*	19	CALPHA	1	1
NM_001024807	chr19:36360709..36370689	*APLP1*	22	CALPHA	1	1
NM_005281	chr1:27720353..27722269	*GPR3*	17	CALPHA	1	1
NM_003105	chr11:121323007..121504463	*SORL1*	88	CALPHA	1	1
NM_004969	chr10:94211444..94333827	*IDE*	27	CALPHA	1	1
NM_001909	chr11:1774022..1782657	*CTSD*	20	CALPHA	1	1
NM_000930	chr8:42032237..42065098	*PLAT*	24	CALPHA	1	1

N. VARIANTS, number of variants. Position is in hg19/GRCh37. Statistical significance p-value < 1.7e^-3^. Corrected p-value, p-value after Bonferroni correction (p-value*29 [number of genes considered in the single-variant association test])

**Table 4:** SKAT TEST.

TRANSCRIPT ID	POSITION	GENE	N.VARIANTS	TEST	P-VALUE	CORRECTED P-VALUE
NM_000371	chr18:29171879..29178899	*TTR*	6	SKAT	0.0234248	0.667
NM_000902	chr3:154797478..154901245	*MME*	40	SKAT	0.0558425	1
NM_003816	chr8:38854521..38962660	*ADAM9*	23	SKAT	0.0986587	1
NM_003183	chr2:9629731..9695906	*ADAM17*	18	SKAT	0.132024	1
NM_002422	chr11:102706685..102714317	*MMP3*	16	SKAT	0.171193	1
NM_003105	chr11:121323007..121504463	*SORL1*	88	SKAT	0.230435	1
NM_001908	chr8:11700101..11725587	*CTSB*	75	SKAT	0.231384	1
NM_015331	chr1:160313330..160328428	*NCSTN*	21	SKAT	0.273587	1
NM_000789	chr17:61554500..61574779	*ACE*	45	SKAT	0.311289	1
NM_012104	chr11:117156543..117186818	*BACE1*	39	SKAT	0.350782	1
NM_005925	chr18:29772759..29800324	*MEP1B*	19	SKAT	0.404511	1
NM_031301	chr15:63569800..63601264	*APH1B*	39	SKAT	0.415623	1
NM_001110	chr15:58888656..59042155	*ADAM10*	26	SKAT	0.416865	1
NM_000239	chr12:69742188..69747889	*LYZ*	5	SKAT	0.454856	1
NM_012105	chr21:42539814..42648229	*BACE2*	88	SKAT	0.465396	1
NM_000099	chr20:23614297..23618571	*CST3*	12	SKAT	0.485137	1
NM_001012964	chr19:51462012..51471329	*KLK6*	11	SKAT	0.586652	1
NM_000014	chr12:9220607..9268549	*A2M*	26	SKAT	0.600323	1
NM_172341	chr19:36237681..36237803	*PSENEN*	2	SKAT	0.61164	1
NM_014693	chr3:183967457..184010734	*ECE2*	32	SKAT	0.808793	1
NM_002332	chr12:57522754..57607023	*LRP1*	111	SKAT	0.813088	1
NM_001163	chr9:72042470..72287163	*APBA1*	41	SKAT	0.815816	1
NM_001024807	chr19:36360709..36370689	*APLP1*	22	SKAT	0.848962	1
NM_004969	chr10:94211444..94333827	*IDE*	27	SKAT	0.868194	1
NM_001397	chr1:21543823..21671981	*ECE1*	51	SKAT	0.894799	1
NM_178011	chr10:68685929..68860827	*LRRTM3*	29	SKAT	0.890455	1
NM_001909	chr11:1774022..1782657	*CTSD*	20	SKAT	0.899719	1
NM_000930	chr8:42032237..42065098	*PLAT*	24	SKAT	0.906314	1
NM_005281	chr1:27720353..27722269	*GPR3*	17	SKAT	0.975572	1

N. VARIANTS, number of variants. Position is in hg19/GRCh37. Statistical significance p-value < 1.7e^-3^. Corrected p-value, p-value after Bonferroni correction (p-value*29 [number of genes considered in the single-variant association test])

## Discussion

The Amyloid cascade hypothesis is the main accepted hypothesis underlying AD pathology. Several genes within the APP-Aβ metabolism pathway have been reported as potential candidate genes for AD. However, coding variability among these has not been extensively investigated. The vast majority of reported studies are based on candidate gene approaches using array-based SNP genotyping and are focused mainly on genes involved in Aβ catabolism (http://www.alzgene.org/). Thus, leaving low frequency and rare coding variants and genes involved in Aβ production largely unexplored.

GWASs and chip-based candidate gene approaches have shown that common and generally non-coding variability within these genes does not play a critical role for AD development. The only exceptions to this general rule are represented by *SORL1* and *ABCA7*, which have been reported associated with late-onset apparently sporadic and familial AD both with GWASs, candidate gene approaches and exome sequencing [25–28].

In this study, we report a screening of genes known to be involved in the APP-Aβ metabolism (APP processing, Aβ production, degradation and clearance). We applied single-marker and gene-based association analyses, to investigate the independent and joint effect of coding variability within these genes in a cohort composed of 332 apparently sporadic and mainly late-onset AD cases and 676 elderly controls from North America and the UK.

In our cohort, genes involved in Aβ degradation and clearance harbor the highest relative frequency of rare and predicted damaging variants (mean = 4.73 rare coding variants/Kbp of coding sequence and 3.16 damaging coding variants/Kbp of coding sequence, respectively). Conversely, genes encoding for proteins regulating Aβ production presented the lowest relative frequency of coding and likely damaging variability (mean = 3.59 rare coding variants/Kbp of coding sequence and 1.5 damaging coding variants/Kbp of coding sequence, respectively) (**S4 and S5 Tables**), suggesting a higher degree of conservation.

In the single-variant association analysis, the main hits were very rare coding variants (MAF 0.009%< MAF<1.4%) with strong effect sizes (1.84<OR<Inf) mapping to genes involved in Aβ extracellular degradation (*ACE*, *TTR*, *CTSB*, *MMP3*), APP trafficking and recycling (*SORL1*), and clearance (*LRP1*). Among the main single-marker hits only 2 genes (*APH1B*, *NCSTN)* were component of the γ secretase complex and therefore pivotal for APP processing. Our study was underpowered for the detection of rare and low frequency variants and these variants were nominally significant after Bonferroni correction (1.9e^-4^<p-value <0.05). Importantly, in our cohort, *TREM2* p.R47H, the second most common risk factor for sporadic AD, has been detected in 6 cases (1.8%) and 4 controls (0.59%) and, given our small sample size, with a MAF = 0.2%, was not significantly associated to AD (p-value = 0.09). Therefore, we suggest that the main variants detected in our study may be functional and warrant a follow up in an extended sample size.

Transthyretin (TTR) is a 55-kDa protein, particularly abundant in the CSF and human plasma, where it transports thyroxin from the peripheral blood circulation to the brain. TTR has been already reported linked to AD dementia. Particularly, it has been hypothesized that TTR may act as a scaffold protein, binding to amyloid and preventing its deposition and aggregation in plaques [29]. Moreover, TTR is a well established biomarker for AD: 1) a decrese in the CSF and serum TTR levels has been associated to an increased AD severity and faster rate of disease progression and 2) 5 SNPs and different *TTR* haplotypes have been related to hippocampal atrophy [30]. By contrast, TTR overexpression decreases Aβ deposition, protects against Aβ plaques formation and improves the cognitive function in different AD mouse model strains [31–32]. Finally, TTR, likewise other well conserved secreted proteins critically involved in dementia (PRP and PGRN), has no homologous proteins (http://string-db.org/), therefore implying that even subtle changes to its epitopes and/or domains may be functionally relevant and phenotypically manifest. In line with this hypothesis, *TTR* is the main hit both in the single-variant analysis and, with only 6 variants detected (4 coding and 2 3´ UTR variants), in the gene-based analysis (SKAT). Thus, suggesting that either the joint effect of the coding and non-coding variability at the *TTR* locus is likely to be functional or that the signal is driven by *TTR* p.T139M, that has a very strong effect size (OR = 6.19, 95% CI = 1.099–63.091).

The second strongest signal in the single variant analysis maps to *ACE* (p.T916M), encoding angiotensin I converting enzyme 1 (ACE1), a zinc metalloprotease, which regulates blood pressure. Multiple lines of evidence have shown that ACE1 may ameliorate the cognitive decline either regulating the cerebral blood flow and/or converting Aβ_42_ to Aβ_40_, a more soluble isoform and its activity is increased in AD brain, in proportion to the parenchymal Aβ load [33–35].

*SORL1* encodes for Sortilin related receptor (SORLA, also known as LR11), which binds to APOE and mediates several intracellular sorting and trafficking functions through a VPS10 (vacuolar protein sorting protein 10) domain [36]. SORLA is highly expressed in the brain and modulates APP recycling through the retromer complex, thereby influencing levels of Aβ [37–38]. A growing body of evidence suggested *SORL1* as an excellent positional and functional candidate for AD [12, 25, 39–42, 25–26]. Moreover, decreased expression and DNA methylation changes at the *SORL1* locus have been reported associated to AD [43–44]. *SORL1* harbors 3 of the top variants identified (p.E270K, p.D2065V and p.R11P). Importantly, p.E270K has been reported in 7 Carribean Hispanic families and *in vitro* studies showed that *SORL1* p.E270K is a functional variant, leading to increased secretion of Aβ, when transfected in HEK293 cell lines and weakening the binding to APP [45]. Therefore, it is plausible that *SORL1* (p.E270K) may increase the susceptibility also for apparently sporadic LOAD. Thus, this finding, with the other main *SORL1* variants detected (p.D2065V and p.R11P) should be further investigated.

Finally, four of the main hits map to the low-density lipoprotein receptor related protein 1 (LRP1) gene. Importantly, LRP1 is a major Aβ clearance receptor in cerebral vascular smooth muscle cells and disturbance of this pathway contributes to Aβ accumulation in the brain [46]. In addition, LRP1 locus was identified as an AD candidate locus in consanguineous Israeli-Arab community [47].

The c-alpha and SKAT tests reported *ECE1* and *LYZ* and *TTR*, respectively, as main genes associated with LOAD, although after the Bonferroni correction, the association was only nominally significant. Very interestingly, the main genes identified both with the single-marker and gene-based analysis play a pivotal role in the cardiovascular system and have been already signaled as potential risk factors for cerebral amyloid angiopathy (CAA) or vascular dementia [48]. First, *ECE1* and *ACE* are key components of the renin-angiotensin cascade, that controls blood pressure [49]. Second, *ECE1*, *ACE* and *TTR* are mostly expressed by endothelial cells in the CNS (http://web.stanford.edu/group/barres_lab/brain_rnaseq.html). Third, acute and chronic hypoxia, through the release of the hypoxia inducible factor 1α (HIF-1α), exerts a critical epigenetic regulation on APP processing and Aβ catabolism key genes. Notably, HIF-1α has been reported to increase γ and β cleavage of APP and impair Aβ degradation and clearance mainly through the down regulation of pivotal proteins such as MME, ECE1 and TTR [50–53].

In summary, our study shows that 1) common coding variability within genes involved in APP-Aβ metabolism does not play a critical role for AD development; 2) genes regulating Aβ production are more conserved than genes playing a key role in Aβ degradation and clearance, thus less frequently involved in sporadic AD; 3) *TTR*, *ACE*, *SORL1*, *CTSB* and *LRP1* harbor rare coding variants with strong effect size, likely to be functional and warrant further investigation in an extended cohort; 4) *ECE1*, *LYZ* and *TTR* play a critical role in the cardiovascular system and the joint effect of their variants may increase the susceptibility to AD. Finally, in concert with previous studies, our results support a potential overlapping biology with shared risk factors between CAA, vascular dementia and AD.

## Supporting Information

S1 Table List of the 29 genes selected in our study. (DOCX)

S2 Table Coding variants detected in APP-Aß metabolism genes in our study. Aa, aminoacid; OR, odds ratio; CI, confidence interval.
(XLSX)

S3 Table Comparison between total variants and relative frequency of variants detected in our study and Exome Variant Server database (EVS) in the European-American cohort. (XLSX)

S4 Table Relative frequency of rare variants in the 29 genes analyzed.Kbp, kilobasepair. (XLSX)

S5 Table Relative frequency of predicted damaging variants in the 29 genes analyzed. The variants have been predicted as damaging by at least 2 out 3 in silico prediction softwares (MUTATION TASTER, SIFT, POLYPHEN2). Kbp, kilobasepair.
(XLSX)

S6 Table **A.** Size-matched negative controls for the gene-based association analysis (c-alpha test). **B.** Size-matched negative controls for the gene-based association analysis (SKAT).
(XLSX)
