# Supplementary material for: Influence of Coding Variability in APP-Aβ Metabolism Genes in Sporadic Alzheimer’s Disease
Source: PLoS One. 2016 Jun 1;11(6):e0150079. doi: 10.1371/journal.pone.0150079 (PMC4889076; doi:10.1371/journal.pone.0150079)
Supplement: S1 Table — (DOCX) [file pone.0150079.s001.docx]

| **GENE** | **TRANSCRIPT** | **NAME** | **FUNCTION** | **REFERENCES** |
| --- | --- | --- | --- | --- |
| *ADAM9* | NM_003816 | ADAM metallopeptidase domain 9 | α-secretase | ^1, 2, 3^ |
| *ADAM10* | NM_001110 | ADAM metallopeptidase domain 10 | α-secretase | ^2, 3, 4,^ |
| *ADAM17* | NM_003183 | ADAM metallopeptidase domain 17 | α-secretase | ^2, 3,^ |
| *BACE1* | NM_012104 | ß -site APP-cleaving enzyme 1 | ß-secretase | ^5, 6^ |
| *BACE2* | NM_012105 | ß -site APP-cleaving enzyme 2 | ß-secretase | ^7, 8,^ |
| *NCSTN* | NM_015331 | nicastrin | γ-secretase | ^9, 10, 11^ |
| *PSENEN (PEN-2)* | NM_172341 | presenilin enhancer γ secretase subunit | γ-secretase | ^11, 12,^ |
| *APH1B* | NM_031301 | APH1B γ secretase subunit | γ-secretase | ^11, 13^ |
| *APLP1* | NM_001024807 | Aß (A4) precursor-like protein 1 | APP agonist cleaved by secretases | ^14, 15^ |
| *APBA1 (MINT1, X11A)* | NM_001163 | Aß (A4) precursor protein-binding, family A, member 1 | It stabilizes APP and inhibits production of proteolytic APP fragments including the Aß | ^16, 17, 18,^ |
| *LRRTM3* | NM_178011 | leucine rich repeat transmembrane neuronal 3 | Aß production | ^19, 20^ |
| *GPR3* | NM_005281 | G-protein coupled receptor 3 | It activates adenylate cyclase and modulates Aß production | ^21^ |
| *TTR* | NM_000371 | transthyretin | Aß deposition | ^22, 23, 24^ |
| *SORL1* | NM_003105 | sortilin-related receptor | APP recycling and vesicles trafficking | ^25, 26, 27, 28, 29, 30, 31, 32, 33, 34^ |
| *ECE1* | NM_001397 | endothelin converting enzyme 1 | intracellular degradation | ^35, 36, 37^ |
| *ECE2* | NM_014693 | endothelin converting enzyme 2 | intracellular degradation | ^35, 36,^ |
| *IDE* | NM_004969 | insulin-degrading enzyme | Intracellular and extracellular degradation | ^38, 39, 40, 41, 42^ |
| *CST3* | NM_000099 | cystatin C | Intracellular and extracellular degradation | ^43, 44, 45, 46^ |
| *CTSB* | NM_001908 | cathepsin B | Intracellular and extracellular degradation | ^43^ |
| *CTSD* | NM_001909 | cathepsin D | intracellular degradation | ^47, 48^ |
| *LYZ* | NM_000239 | lysozyme | intracellular degradation | ^49^ |
| *MME (Neprylisin)* | NM_000902 | membrane metallo-endopeptidase | extracellular degradation | ^50, 51, 52, 53, 54^ |
| *ACE* | NM_000789 | angiotensin I converting enzyme | extracellular degradation | ^55, 56, 57^ |
| *MMP3* | NM_002422 | matrix metallopeptidase | extracellular degradation | ^58, 59, 60, 61^ |
| *A2M* | NM_000014 | α-2-macroglobulin | extracellular degradation | ^62, 63, 64, 65, 66^ |
| *PLAT* | NM_000930 | plasminogen activator, tissue | extracellular degradation | ^67, 68, 69, 70^ |
| *MEP1B* | NM_005925 | meprin A, β | extracellular degradation | ^71, 72, 73,^ |
| *KLK6* | NM_001012964 | kallikrein-related peptidase peptidase 6 | extracellular degradation | ^74^ |
| *LRP1* | NM_002332 | Low density lipoprotein receptor-related protein 1 | extracellular clearance | ^75, 76^ |

**S1 Table. List of the 29 genes selected in our study**

**Bioinformatic**

Each of the samples in our dataset consisted of paired-end 100 base pair reads. We used the Burrows-Wheeler Aligner (BWA)^77^ to map the reads to the human genome (hg19/GRCh37). Following read mapping, we used SAMtools ^78^, Picard (<http://picard.sourceforge.net>), and the Genome Analysis Toolkit (GATK) ^79,80^ to refine the resulting alignments by removing duplicates, performing realignment around InDels, and recalibrating base quality scores. We then used the GATK’s UnifiedGenotyper to identify sequence variants, and subsequently filtered the variants and recalibrated variant quality scores ^79^. Our final dataset consisted of variant call format (VCF) files containing variants that passed all filters. Since our dataset consisted of a mix of exomes captured using different kits, and whole genome sequences, we employed a highly conservative approach to variant selection to increase our confidence that analyzed variants are true positives. We limited our dataset of variants to only those genomic regions we expected to have been sequenced in each of the exomes (based on capture probes used for exome library preparation) and whole genomes. Next, we compiled a list of all the variants present in at least a single sample. We examined each of the variants from the list of total variants in each sample, whether or not the variant was called by the GATK, and reassigned the genotype for that variant according to the following criteria: 1) If the variant was called by the GATK and passed all filters, we used the GATK genotype; 2) If no variant was called at the genomic position in question, we returned to the raw VCF file and if there were reads containing the variant, but the variant was not called because of failing filters or because only a small number of reads contain the variant, we set the genotype to missing for the sample and 3) if all the reads at this position for the sample indicated reference alleles, we set the genotype to homozygous reference. Resulting sequence files were converted to Plink format ^81^ using VCFTools ^82^. Lastly, we removed all variants not in our pre-defined list of candidate genes (*A2M* [NM_000014], *ACE* [NM_000789], *ADAM9* [NM_003816], *ADAM10* [NM_001110], *ADAM17* [NM_003183], *APBA1* [NM_001163], *APH1B* [NM_031301], *APLP1* [NM_001024807], *BACE1* [NM_012104], *BACE2* [NM_012105], *CST3* [NM_000099], *CTSB* [NM_001908], *CTSD* [NM_001909], *ECE1* [NM_001397], *ECE2* [NM_014693], *GPR3* [NM_005281], *IDE* [NM_004969], *LRP1* [NM_002332], *KLK6* [NM_001012964], *LRRTM3* [NM_178011], *LYZ* [NM_000239], *MEP1B* [NM_005925], *MME* [NM_000902], *MMP3* [NM_002422], *NCSTN* [NM_015331] , *PLAT* [NM_000930], *PSENEN* [NM_172341], *SORL1* [NM_003105], *TTR* [NM_000371]) (**Table S1**). Remaining variants were annotated using ANNOVAR ^83^. Each variant was annotated with gene information (gene name, transcript ID, and transcript and protein positions of the variant), genomic location (exon, intron, UTR, intergenic, etc.), one or more variant classes (5’-UTR, 3’-UTR, intergenic, intronic, splice site, nonsynonymous, stop-gain, stop-loss, or synonymous), the 1000 Genomes minor allele frequency ^84^, dbSNP identifier^85^, and PolyPhen-2 ^86^ and SIFT ^87^ functional predictions.

1. Moss, M. L. *et al.* ADAM9 inhibition increases membrane activity of ADAM10 and controls α-secretase processing of amyloid precursor protein. *J. Biol. Chem.* **286,** 40443–40451 (2011).

2. Allinson, T. M. J., Parkin, E. T., Turner, A. J. & Hooper, N. M. ADAMs family members as amyloid precursor protein alpha-secretases. *J. Neurosci. Res.* **74,** 342–352 (2003).

3. Asai, M. *et al.* Putative function of ADAM9, ADAM10, and ADAM17 as APP alpha-secretase. *Biochem. Biophys. Res. Commun.* **301,** 231–235 (2003).

4. Suh, J. *et al.* ADAM10 missense mutations potentiate β-amyloid accumulation by impairing prodomain chaperone function. *Neuron* **80,** 385–401 (2013).

5. Sun, X., Tong, Y., Qing, H., Chen, C.-H. & Song, W. Increased BACE1 maturation contributes to the pathogenesis of Alzheimer’s disease in Down syndrome. *FASEB J. Off. Publ. Fed. Am. Soc. Exp. Biol.* **20,** 1361–1368 (2006).

6. Willem, M., Lammich, S. & Haass, C. Function, regulation and therapeutic properties of beta-secretase (BACE1). *Semin. Cell Dev. Biol.* **20,** 175–182 (2009).

7. Myllykangas, L. *et al.* Chromosome 21 BACE2 haplotype associates with Alzheimer’s disease: a two-stage study. *J. Neurol. Sci.* **236,** 17–24 (2005).

8. Farzan, M., Schnitzler, C. E., Vasilieva, N., Leung, D. & Choe, H. BACE2, a beta -secretase homolog, cleaves at the beta site and within the amyloid-beta region of the amyloid-beta precursor protein. *Proc. Natl. Acad. Sci. U. S. A.* **97,** 9712–9717 (2000).

9. Capell, A. *et al.* Nicastrin interacts with gamma-secretase complex components via the N-terminal part of its transmembrane domain. *J. Biol. Chem.* **278,** 52519–52523 (2003).

10. Shirotani, K. *et al.* Gamma-secretase activity is associated with a conformational change of nicastrin. *J. Biol. Chem.* **278,** 16474–16477 (2003).

11. Edbauer, D. *et al.* Reconstitution of gamma-secretase activity. *Nat. Cell Biol.* **5,** 486–488 (2003).

12. Steiner, H. *et al.* PEN-2 is an integral component of the gamma-secretase complex required for coordinated expression of presenilin and nicastrin. *J. Biol. Chem.* **277,** 39062–39065 (2002).

13. Serneels, L. *et al.* gamma-Secretase heterogeneity in the Aph1 subunit: relevance for Alzheimer’s disease. *Science* **324,** 639–642 (2009).

14. Yanagida, K. *et al.* The 28-amino acid form of an APLP1-derived Abeta-like peptide is a surrogate marker for Abeta42 production in the central nervous system. *EMBO Mol. Med.* **1,** 223–235 (2009).

15. Neumann, S. *et al.* Amyloid precursor-like protein 1 influences endocytosis and proteolytic processing of the amyloid precursor protein. *J. Biol. Chem.* **281,** 7583–7594 (2006).

16. Matos, M. F. *et al.* Autoinhibition of Mint1 adaptor protein regulates amyloid precursor protein binding and processing. *Proc. Natl. Acad. Sci. U. S. A.* **109,** 3802–3807 (2012).

17. Xie, Z., Romano, D. M. & Tanzi, R. E. RNA interference-mediated silencing of X11alpha and X11beta attenuates amyloid beta-protein levels via differential effects on beta-amyloid precursor protein processing. *J. Biol. Chem.* **280,** 15413–15421 (2005).

18. Lee, J.-H. *et al.* The neuronal adaptor protein X11alpha reduces Abeta levels in the brains of Alzheimer’s APPswe Tg2576 transgenic mice. *J. Biol. Chem.* **278,** 47025–47029 (2003).

19. Reitz, C., Conrad, C., Roszkowski, K., Rogers, R. S. & Mayeux, R. Effect of genetic variation in LRRTM3 on risk of Alzheimer disease. *Arch. Neurol.* **69,** 894–900 (2012).

20. Majercak, J. *et al.* LRRTM3 promotes processing of amyloid-precursor protein by BACE1 and is a positional candidate gene for late-onset Alzheimer’s disease. *Proc. Natl. Acad. Sci. U. S. A.* **103,** 17967–17972 (2006).

21. Thathiah, A. *et al.* The orphan G protein-coupled receptor 3 modulates amyloid-beta peptide generation in neurons. *Science* **323,** 946–951 (2009).

22. Costa, R., Gonçalves, A., Saraiva, M. J. & Cardoso, I. Transthyretin binding to A-Beta peptide--impact on A-Beta fibrillogenesis and toxicity. *FEBS Lett.* **582,** 936–942 (2008).

23. Choi, S. H. *et al.* Accelerated Abeta deposition in APPswe/PS1deltaE9 mice with hemizygous deletions of TTR (transthyretin). *J. Neurosci. Off. J. Soc. Neurosci.* **27,** 7006–7010 (2007).

24. Schwarzman, A. L. *et al.* Transthyretin sequesters amyloid beta protein and prevents amyloid formation. *Proc. Natl. Acad. Sci. U. S. A.* **91,** 8368–8372 (1994).

25. Feulner, T. M. *et al.* Examination of the current top candidate genes for AD in a genome-wide association study. *Mol. Psychiatry* **15,** 756–766 (2010).

26. Lambert, J. C. *et al.* Meta-analysis of 74,046 individuals identifies 11 new susceptibility loci for Alzheimer’s disease. *Nat. Genet.* **45,** 1452–1458 (2013).

27. Rogaeva, E. *et al.* The neuronal sortilin-related receptor SORL1 is genetically associated with Alzheimer disease. *Nat. Genet.* **39,** 168–177 (2007).

28. Miyashita, A. *et al.* SORL1 is genetically associated with late-onset Alzheimer’s disease in Japanese, Koreans and Caucasians. *PloS One* **8,** e58618 (2013).

29. Tan, E. K. *et al.* SORL1 haplotypes modulate risk of Alzheimer’s disease in Chinese. *Neurobiol. Aging* **30,** 1048–1051 (2009).

30. Lee, J. H. *et al.* The association between genetic variants in SORL1 and Alzheimer disease in an urban, multiethnic, community-based cohort. *Arch. Neurol.* **64,** 501–506 (2007).

31. Pottier, C. *et al.* High frequency of potentially pathogenic SORL1 mutations in autosomal dominant early-onset Alzheimer disease. *Mol. Psychiatry* **17,** 875–879 (2012).

32. Yamazaki, H. *et al.* Elements of neural adhesion molecules and a yeast vacuolar protein sorting receptor are present in a novel mammalian low density lipoprotein receptor family member. *J. Biol. Chem.* **271,** 24761–24768 (1996).

33. Andersen, O. M. *et al.* Neuronal sorting protein-related receptor sorLA/LR11 regulates processing of the amyloid precursor protein. *Proc. Natl. Acad. Sci. U. S. A.* **102,** 13461–13466 (2005).

34. Offe, K. *et al.* The lipoprotein receptor LR11 regulates amyloid beta production and amyloid precursor protein traffic in endosomal compartments. *J. Neurosci. Off. J. Soc. Neurosci.* **26,** 1596–1603 (2006).

35. Hamilton, G. *et al.* The role of ECE1 variants in cognitive ability in old age and Alzheimer’s disease risk. *Am. J. Med. Genet. Part B Neuropsychiatr. Genet. Off. Publ. Int. Soc. Psychiatr. Genet.* **159B,** 696–709 (2012).

36. Natunen, T. *et al.* Genetic analysis of genes involved in amyloid-β degradation and clearance in Alzheimer’s disease. *J. Alzheimers Dis. JAD* **28,** 553–559 (2012).

37. Cousin, E. *et al.* No replication of genetic association between candidate polymorphisms and Alzheimer’s disease. *Neurobiol. Aging* **32,** 1443–1451 (2011).

38. Zou, F. *et al.* Gene expression levels as endophenotypes in genome-wide association studies of Alzheimer disease. *Neurology* **74,** 480–486 (2010).

39. Tsuda, M., Kobayashi, T., Matsuo, T. & Aigaki, T. Insulin-degrading enzyme antagonizes insulin-dependent tissue growth and Abeta-induced neurotoxicity in Drosophila. *FEBS Lett.* **584,** 2916–2920 (2010).

40. Carrasquillo, M. M. *et al.* Concordant association of insulin degrading enzyme gene (IDE) variants with IDE mRNA, Abeta, and Alzheimer’s disease. *PloS One* **5,** e8764 (2010).

41. Vepsäläinen, S. *et al.* Increased expression of Abeta degrading enzyme IDE in the cortex of transgenic mice with Alzheimer’s disease-like neuropathology. *Neurosci. Lett.* **438,** 216–220 (2008).

42. Kim, M. *et al.* Decreased catalytic activity of the insulin-degrading enzyme in chromosome 10-linked Alzheimer disease families. *J. Biol. Chem.* **282,** 7825–7832 (2007).

43. Sun, B. *et al.* Cystatin C-cathepsin B axis regulates amyloid beta levels and associated neuronal deficits in an animal model of Alzheimer’s disease. *Neuron* **60,** 247–257 (2008).

44. Sundelöf, J. *et al.* Serum cystatin C and the risk of Alzheimer disease in elderly men. *Neurology* **71,** 1072–1079 (2008).

45. Kaeser, S. A. *et al.* Cystatin C modulates cerebral beta-amyloidosis. *Nat. Genet.* **39,** 1437–1439 (2007).

46. Mi, W. *et al.* Cystatin C inhibits amyloid-beta deposition in Alzheimer’s disease mouse models. *Nat. Genet.* **39,** 1440–1442 (2007).

47. Papassotiropoulos, A. *et al.* Genetic polymorphism of cathepsin D is strongly associated with the risk for developing sporadic Alzheimer’s disease. *Neurosci. Lett.* **262,** 171–174 (1999).

48. Hoffman, K. B., Bi, X., Pham, J. T. & Lynch, G. Beta-amyloid increases cathepsin D levels in hippocampus. *Neurosci. Lett.* **250,** 75–78 (1998).

49. Luo, J., Wärmländer, S. K. T. S., Gräslund, A. & Abrahams, J. P. Human lysozyme inhibits the in vitro aggregation of Aβ peptides, which in vivo are associated with Alzheimer’s disease. *Chem. Commun. Camb. Engl.* **49,** 6507–6509 (2013).

50. Miners, S. *et al.* Genetic variation in MME in relation to neprilysin protein and enzyme activity, Aβ levels, and Alzheimer’s disease risk. *Int. J. Mol. Epidemiol. Genet.* **3,** 30–38 (2012).

51. Helisalmi, S. *et al.* Polymorphisms in neprilysin gene affect the risk of Alzheimer’s disease in Finnish patients. *J. Neurol. Neurosurg. Psychiatry* **75,** 1746–1748 (2004).

52. Mohajeri, M. H. *et al.* Anti-amyloid activity of neprilysin in plaque-bearing mouse models of Alzheimer’s disease. *FEBS Lett.* **562,** 16–21 (2004).

53. Iwata, N. *et al.* Presynaptic localization of neprilysin contributes to efficient clearance of amyloid-beta peptide in mouse brain. *J. Neurosci. Off. J. Soc. Neurosci.* **24,** 991–998 (2004).

54. Iwata, N. *et al.* Metabolic regulation of brain Abeta by neprilysin. *Science* **292,** 1550–1552 (2001).

55. Zou, K. *et al.* Abeta42-to-Abeta40- and angiotensin-converting activities in different domains of angiotensin-converting enzyme. *J. Biol. Chem.* **284,** 31914–31920 (2009).

56. Hu, J., Igarashi, A., Kamata, M. & Nakagawa, H. Angiotensin-converting enzyme degrades Alzheimer amyloid beta-peptide (A beta ); retards A beta aggregation, deposition, fibril formation; and inhibits cytotoxicity. *J. Biol. Chem.* **276,** 47863–47868 (2001).

57. Hemming, M. L. & Selkoe, D. J. Amyloid beta-protein is degraded by cellular angiotensin-converting enzyme (ACE) and elevated by an ACE inhibitor. *J. Biol. Chem.* **280,** 37644–37650 (2005).

58. Mlekusch, R. & Humpel, C. Matrix metalloproteinases-2 and -3 are reduced in cerebrospinal fluid with low beta-amyloid1-42 levels. *Neurosci. Lett.* **466,** 135–138 (2009).

59. Helbecque, N., Cottel, D., Hermant, X. & Amouyel, P. Impact of the matrix metalloproteinase MMP-3 on dementia. *Neurobiol. Aging* **28,** 1215–1220 (2007).

60. Saarela, M. S. *et al.* Interaction between matrix metalloproteinase 3 and the epsilon4 allele of apolipoprotein E increases the risk of Alzheimer’s disease in Finns. *Neurosci. Lett.* **367,** 336–339 (2004).

61. Yoshiyama, Y., Asahina, M. & Hattori, T. Selective distribution of matrix metalloproteinase-3 (MMP-3) in Alzheimer’s disease brain. *Acta Neuropathol. (Berl.)* **99,** 91–95 (2000).

62. Qiu, W. Q. *et al.* Degradation of amyloid beta-protein by a serine protease-alpha2-macroglobulin complex. *J. Biol. Chem.* **271,** 8443–8451 (1996).

63. Saunders, A. J. *et al.* Genetic association of Alzheimer’s disease with multiple polymorphisms in alpha-2-macroglobulin. *Hum. Mol. Genet.* **12,** 2765–2776 (2003).

64. Zappia, M. *et al.* Genetic association of alpha2-macroglobulin polymorphisms with AD in southern Italy. *Neurology* **59,** 756–758 (2002).

65. Pirskanen, M. *et al.* An association between a subset of Finnish late-onset Alzheimer’s disease and alpha2-macroglobulin. *Neurogenetics* **3,** 171–172 (2001).

66. Verpillat, P. *et al.* Alpha2-macroglobulin gene and Alzheimer’s disease: confirmation of association by haplotypes analyses. *Ann. Neurol.* **48,** 400–402 (2000).

67. Lee, J.-Y. *et al.* Upregulation of tPA/plasminogen proteolytic system in the periphery of amyloid deposits in the Tg2576 mouse model of Alzheimer’s disease. *Neurosci. Lett.* **423,** 82–87 (2007).

68. Medina, M. G. *et al.* Tissue plasminogen activator mediates amyloid-induced neurotoxicity via Erk1/2 activation. *EMBO J.* **24,** 1706–1716 (2005).

69. Melchor, J. P., Pawlak, R. & Strickland, S. The tissue plasminogen activator-plasminogen proteolytic cascade accelerates amyloid-beta (Abeta) degradation and inhibits Abeta-induced neurodegeneration. *J. Neurosci. Off. J. Soc. Neurosci.* **23,** 8867–8871 (2003).

70. Kingston, I. B., Castro, M. J. & Anderson, S. In vitro stimulation of tissue-type plasminogen activator by Alzheimer amyloid beta-peptide analogues. *Nat. Med.* **1,** 138–142 (1995).

71. Bien, J. *et al.* The metalloprotease meprin β generates amino terminal-truncated amyloid β peptide species. *J. Biol. Chem.* **287,** 33304–33313 (2012).

72. Jefferson, T. *et al.* The substrate degradome of meprin metalloproteases reveals an unexpected proteolytic link between meprin β and ADAM10. *Cell. Mol. Life Sci. CMLS* **70,** 309–333 (2013).

73. Jefferson, T. *et al.* Metalloprotease meprin beta generates nontoxic N-terminal amyloid precursor protein fragments in vivo. *J. Biol. Chem.* **286,** 27741–27750 (2011).

74. Ashby, E. L., Kehoe, P. G. & Love, S. Kallikrein-related peptidase 6 in Alzheimer’s disease and vascular dementia. *Brain Res.* **1363,** 1–10 (2010).

75. Kanekiyo, T., Liu, C.-C., Shinohara, M., Li, J. & Bu, G. LRP1 in brain vascular smooth muscle cells mediates local clearance of Alzheimer’s amyloid-β. *J. Neurosci. Off. J. Soc. Neurosci.* **32,** 16458–16465 (2012).

76. Farrer, L. A. *et al.* Identification of multiple loci for Alzheimer disease in a consanguineous Israeli-Arab community. *Hum. Mol. Genet.* **12,** 415–422 (2003).

77. Li, H. & Durbin, R. Fast and accurate short read alignment with Burrows-Wheeler transform. *Bioinforma. Oxf. Engl.* **25,** 1754–1760 (2009).

78. Li, H. *et al.* The Sequence Alignment/Map format and SAMtools. *Bioinforma. Oxf. Engl.* **25,** 2078–2079 (2009).

79. DePristo, M. A. *et al.* A framework for variation discovery and genotyping using next-generation DNA sequencing data. *Nat. Genet.* **43,** 491–498 (2011).

80. McKenna, A. *et al.* The Genome Analysis Toolkit: a MapReduce framework for analyzing next-generation DNA sequencing data. *Genome Res.* **20,** 1297–1303 (2010).

81. Purcell, S. *et al.* PLINK: a tool set for whole-genome association and population-based linkage analyses. *Am. J. Hum. Genet.* **81,** 559–575 (2007).

82. Danecek, P. *et al.* The variant call format and VCFtools. *Bioinforma. Oxf. Engl.* **27,** 2156–2158 (2011).

83. Wang, K., Li, M. & Hakonarson, H. ANNOVAR: functional annotation of genetic variants from high-throughput sequencing data. *Nucleic Acids Res.* **38,** e164 (2010).

84. 1000 Genomes Project Consortium *et al.* An integrated map of genetic variation from 1,092 human genomes. *Nature* **491,** 56–65 (2012).

85. Sherry, S. T. *et al.* dbSNP: the NCBI database of genetic variation. *Nucleic Acids Res.* **29,** 308–311 (2001).

86. Adzhubei, I. A. *et al.* A method and server for predicting damaging missense mutations. *Nat. Methods* **7,** 248–249 (2010).

87. Kumar, P., Henikoff, S. & Ng, P. C. Predicting the effects of coding non-synonymous variants on protein function using the SIFT algorithm. *Nat. Protoc.* **4,** 1073–1081 (2009).
